# Supplementary material for: Hepatic Transcriptomics Reveals Reduced Lipogenesis in High-Salt Diet Mice
Source: Genes (Basel). 2023 Apr 24;14(5):966. doi: 10.3390/genes14050966 (PMC10218062; doi:10.3390/genes14050966)
Supplement: Supplementary file 1 [file genes-14-00966-s001.zip › Supplementary Figure S3_Pie chart of DEGs and qPCR verification of six more DEGs within the up-regulation group.pdf]

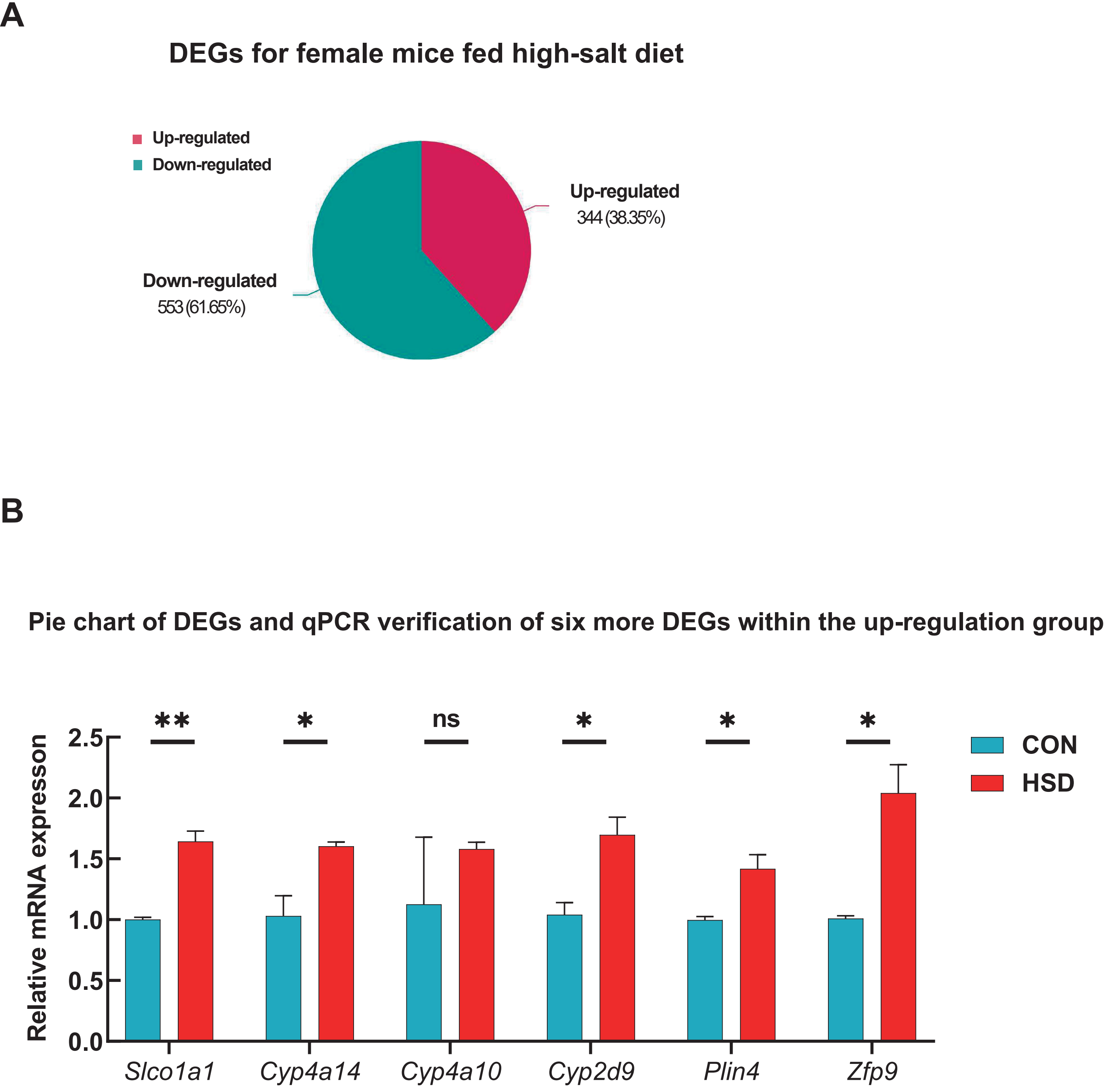
A
DEGs for female mice fed high-salt diet
Up-regulated
Down-regulated
Up-regulated
344 (38.35%)
Down-regulated
553 (61.65%)
B
Pie chart of DEGs and qPCR verification of six more DEGs within the up-regulation group
**
*
ns
*
*
*
CON
HSD
Relative mRNA expresson
0.0
0.5
1.0
1.5
2.0
2.5
Slco1a1
Cyp4a14
Cyp4a10
Cyp2d9
Plin4
Zfp9
