# Supplementary material for: Hepatic Transcriptomics Reveals Reduced Lipogenesis in High-Salt Diet Mice
Source: Genes (Basel). 2023 Apr 24;14(5):966. doi: 10.3390/genes14050966 (PMC10218062; doi:10.3390/genes14050966)
Supplement: Supplementary file 1 [file genes-14-00966-s001.zip › Supplementary Tables S1_The primers of DEGs validated by RT-qPCR analysis.pdf]

Supplementary Table S1 The primers of DEGs validated by RT-qPCR analysis

Gene	Accession Number	Sequences
Gapdh	NM_008084.3	F-5‘-aggtcggtgtgaacggatttg-3'<br>R-5‘-tgtagaccatgtagttgaggtca -3'
Acly	NM_134037	F-5‘- accctttcactggggatcaca -3'<br>R-5‘- gacagggatcaggatttccttg -3'
Fasn	NM_007988.3	F-5‘-ggaggtggtgatagccggtat-3'<br>R-5‘-tgggtaatccatagagcccag-3'
Scd1	NM_009127.4	F-5‘-ttcttgcgatacactctggtgc-3'<br>R-5‘-cgggattgaatgttcttgtcgt-3'
Cd36	NM_001159556	F-5‘- atgggctgtgatcggaactg-3'<br>R-5‘- gtcttcccaataagcatgtctcc-3'
Acaca	NM_133360	F-5‘- atgggcggaatggtctctttc -3'<br>R-5‘- tggggaccttgtcttcatcat -3'
Acot1	NM_012006	F-5‘- atacccctgtgactatcctga-3'<br>R-5‘- caaacactcactacccaactgt-3'
G6pc	NM_008061.4	F-5‘-cgactcgctatctccaagtga-3'<br>R-5‘-gttgaaccagtctccgacca-3'
Pck1	NM_011044.3	F-5‘-ctgcataacggtctggacttc-3'<br>R-5‘-cagcaactgcccgtactcc-3'
Cyp4a10	NM_010011.3	F-5‘- ttccctgatggacgctcttta-3'<br>R-5‘- gcaaacctggaagggtcaaac-3'
Cyp4a14	NM_007822.2	F-5‘-tttagccctacaaggtacttgga-3'<br>R-5‘-gcagccactgccttcgtaa-3'
Cyp2d9	NM_010006.2	F-5‘-gctgaaagatgagtctgtctgg-3'<br>R-5‘-tggtctcgtaccacagcaca-3'
Zfp9	NM_011763.2	F-5‘-cgtgacattaagggatgttgct-3'<br>R-5‘-ccacacgataacccacagaga-3'
Plin4	NM_001372234.1	F-5‘-gtgtccaccaactcacagatg-3'<br>R-5‘-ggaccattccttttgcagcat-3'
Slco1a1	NM_013797.5	F-5‘-gtgcatacctagccaaatcact-3'<br>R-5‘-ccaggcccataaccacacatc-3'

Rdh9	NM_153133.2	F-5‘-gggccgagtatctttgtgtgg -3'<br>R-5‘-tcagggaagtccaatagagaagg-3'
Ugt2b37	NM_053215.3	F-5‘- tggggccaacagtgtctatga -3'<br>R-5‘- gcattgagcacatctgacctt-3'
Rdh11	NM_001362269.1	F-5‘- tgggcaggattcacttccata-3'<br>R-5‘- gaggagtaccgcgtcaattca -3'
Cyp3a41b	NM_001105159.1	F-5‘- aaagccgcctcgattctaagc-3'<br>R-5‘- actacatcccgtggtacaacc-3'
Ugt1a9	NM_201644.2	F-5‘- tctcgctcccatcagtaatctt -3'<br>R-5‘- tggttccacactctctccttg-3'
Aldh1a7	NM_011921.2	F-5‘- acttggaagttaggccctgc-3'<br>R-5‘- tgtgaaggacactttgtcgatg-3'
Acsl5	NM_027976.2	F-5‘- tcctgacgtttggaacggc-3'<br>R-5‘- ctccctcaatccccacagac-3'
Cyp7a1	NM_007824.3	F-5‘- gggattgctgtggtagtgagc-3'<br>R-5‘- ggtatggaatcaacccgttgtc-3'
Fabp5	NM_001272097.1	F-5‘- tgaaagagctaggagtaggactg-3'<br>R-5‘- ctctcggttttgaccgtgatg-3'
Ehhadh	NM_023737.3	F-5‘- atggctgagtatctgaggctg-3'<br>R-5‘- ggtccaaactagctttctggag -3'
Me1	NM_001198933.1	F-5‘-gtcgtgcatctctcacagaag -3'<br>R-5‘-tgagggcagttggttttatcttt -3'
Plin2	NM_001403711.1	F-5‘-gaccttgtgtcctccgcttat -3'<br>R-5‘- caaccgcaatttgtggctc-3'
Aqp8	NM_001109045.1	F-5‘- tgtgtagtatggacctacctgag-3'<br>R-5‘- accgatagacatccgatgaagat -3'
Acnat2	NM_001356289.1	F-5‘- aagcgggaacagattcaagaag-3'<br>R-5‘- acgaaattcaactagaccccca-3'
Abcb1a	NM_011076.3	F-5‘- cagcagtcagtgtgcttacaa -3'<br>R-5‘- atggctcttttatcggcctca-3'
Mylip	NM_153789.3	F-5‘- atgctgtgctatgtgacgagg -3'<br>R-5‘- tcgatgatccctagacgcctg -3'

Sort1	NM_001271599.1	F-5‘- cccggacttcatcgccaag -3'<br>R-5‘- aggacgagaataaccccagtg-3'
Lipg	NM_010720.3	F-5‘- atgcgaaacacggttttcctg -3'<br>R-5‘- gtagctggtactccagtggg -3'
