# Supplementary material for: Hepatic Transcriptomics Reveals Reduced Lipogenesis in High-Salt Diet Mice
Source: Genes (Basel). 2023 Apr 24;14(5):966. doi: 10.3390/genes14050966 (PMC10218062; doi:10.3390/genes14050966)
Supplement: Supplementary file 1 [file genes-14-00966-s001.zip › Supplementary Tables S2_Statistic analysis of clean reads for transcriptome sequencing.pdf]

Supplementary Table S2. Statistic analysis of clean reads for transcriptome sequencing

Library	CONL2	CONL3	CONL4	HSL6	HSL8	HSL9
Total Reads	44,301,190	45,862,628	46,727,208	44,287,150	46,439,024	44,861,682
Mapped Reads	43,025,413	44,261,179	45,408,987	42,991,091	45,038,393	43,511,441
Mapping Rate	0.9712	0.9651	0.9718	0.9707	0.9698	0.9699
UnMapped Reads	1,275,777	1,601,449	1,318,221	1,296,059	1,400,631	1,350,241
MultiMap Reads	3,818,347	4,383,971	4,006,561	3,455,620	3,948,891	3,876,286
MultiMap Rate	0.0862	0.0956	0.0857	0.078	0.085	0.0864
