# Supplementary figures and images for: Hepatic Transcriptomics Reveals Reduced Lipogenesis in High-Salt Diet Mice
Source: Genes (Basel). 2023 Apr 24;14(5):966. doi: 10.3390/genes14050966 (PMC10218062; doi:10.3390/genes14050966)

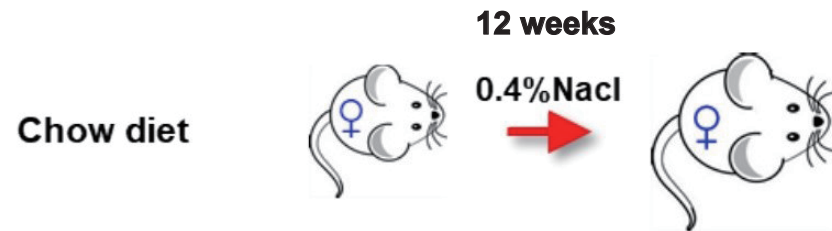

**6 weeks old  
c57BL/6J**

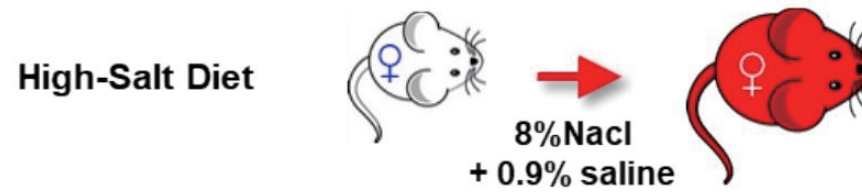

Supplement: Supplementary file 1 [file genes-14-00966-s001.zip › Supplementary Figure S1_Schematic description of animal experiments.pdf]

PCA 3D figure

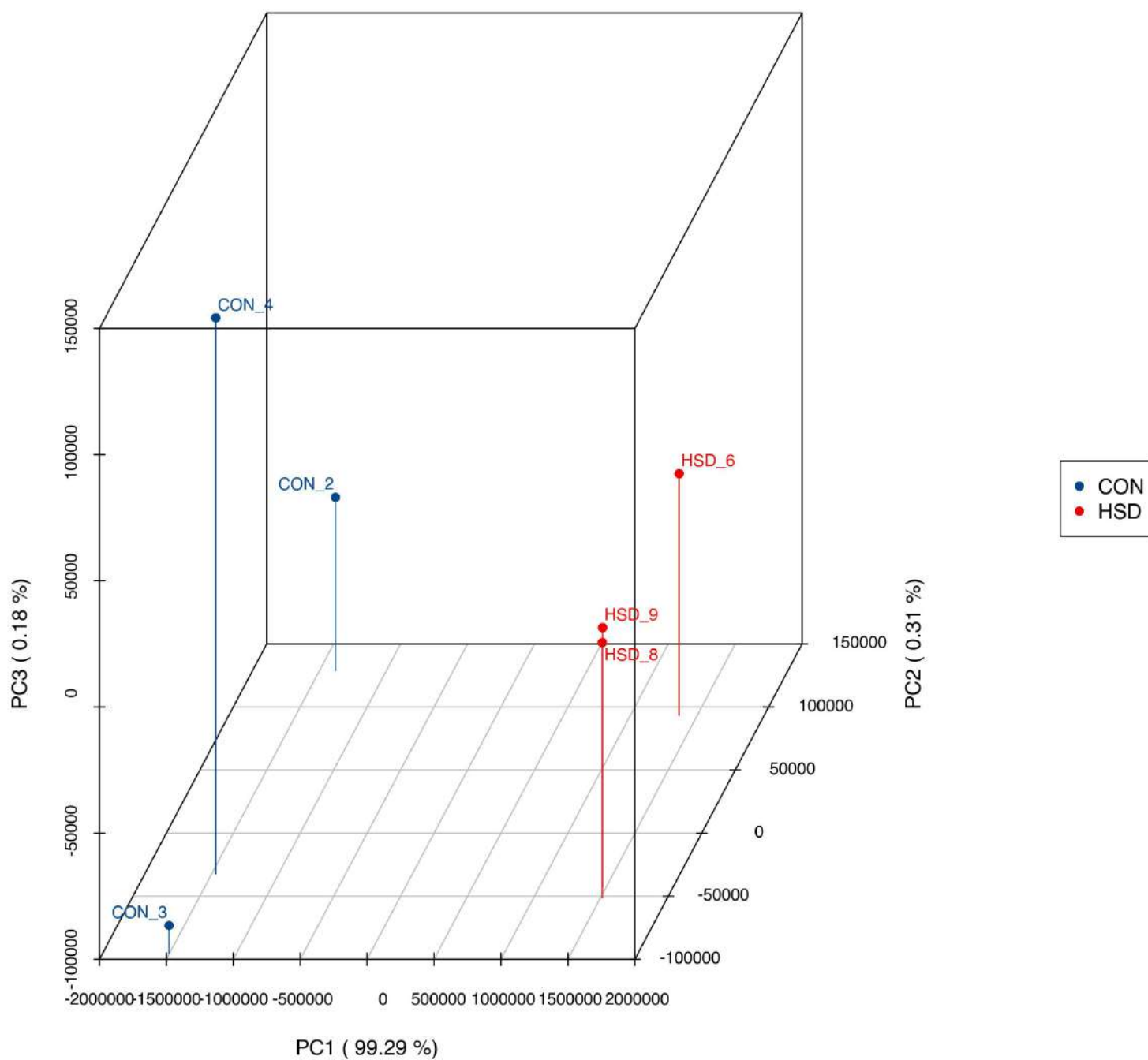

Supplement: Supplementary file 1 [file genes-14-00966-s001.zip › Supplementary Figure S2_Principal component analysis (PCA) for clustering.pdf]

A

## GO analysis of up-regulated DEGs

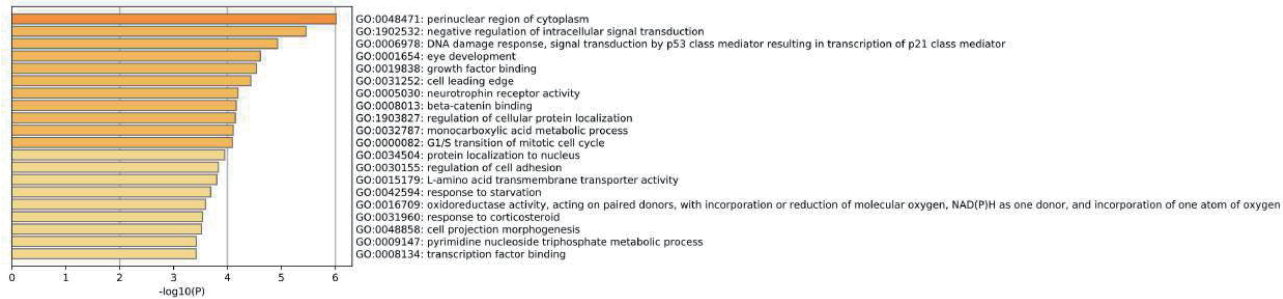

B

## GO analysis of down-regulated DEGs

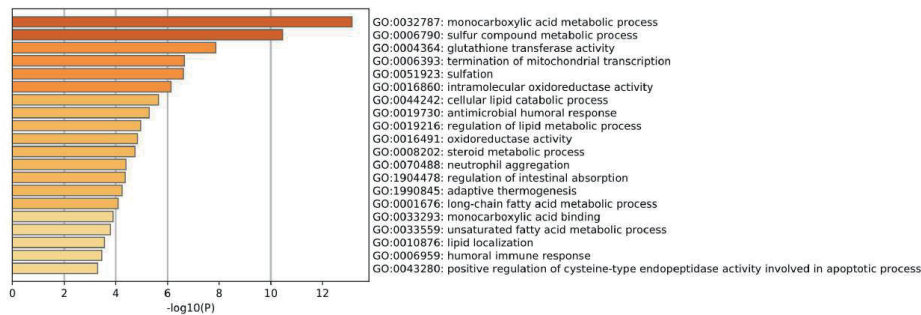

Supplement: Supplementary file 1 [file genes-14-00966-s001.zip › Supplementary Figure S4_ GO analysis of up-and down-regulated DEGs.pdf]

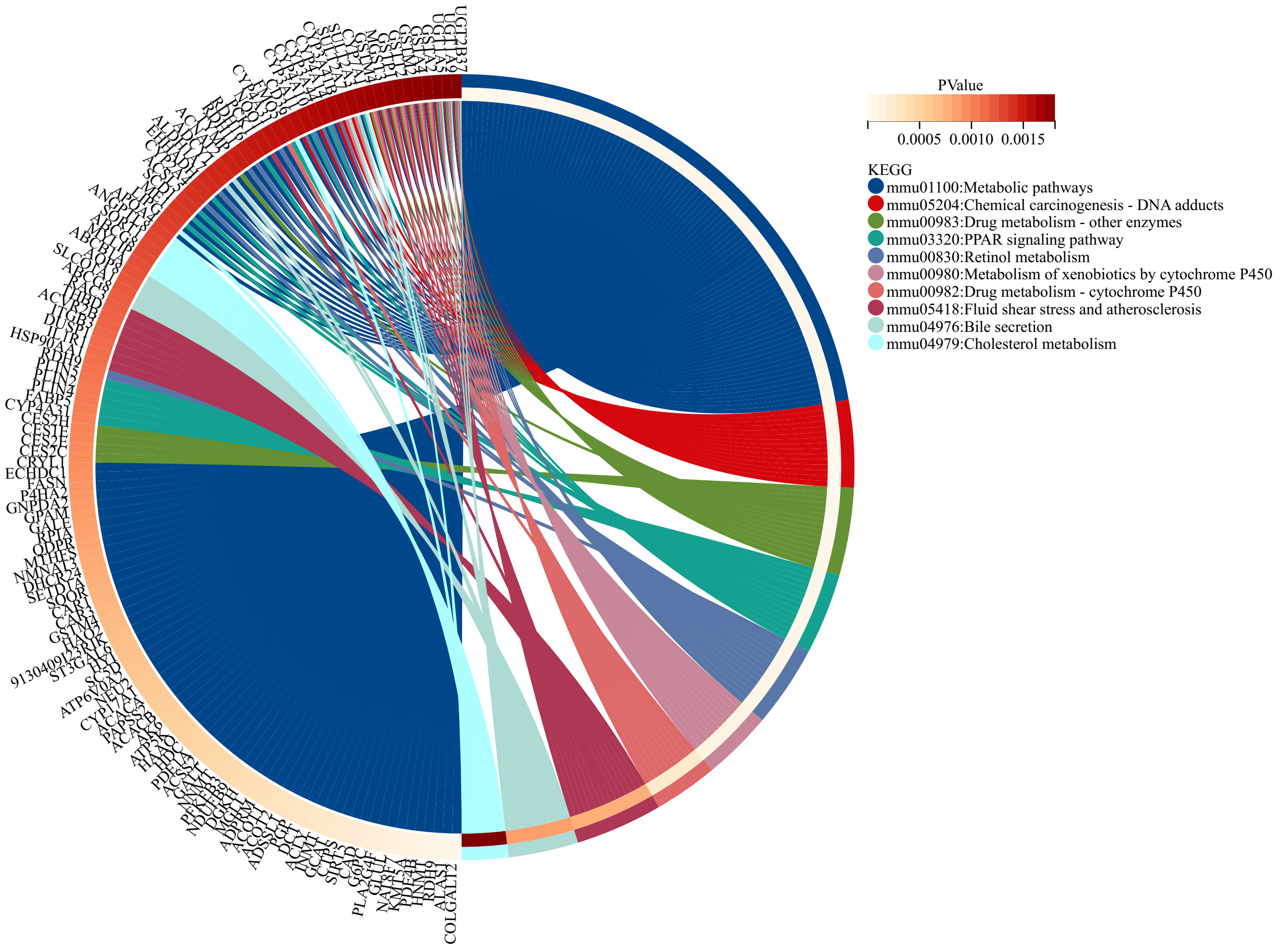

Supplement: Supplementary file 1 [file genes-14-00966-s001.zip › Supplementary Figure S5_Circus plot of top 10 KEGG pathways..pdf]

## A PPAR signaling pathway

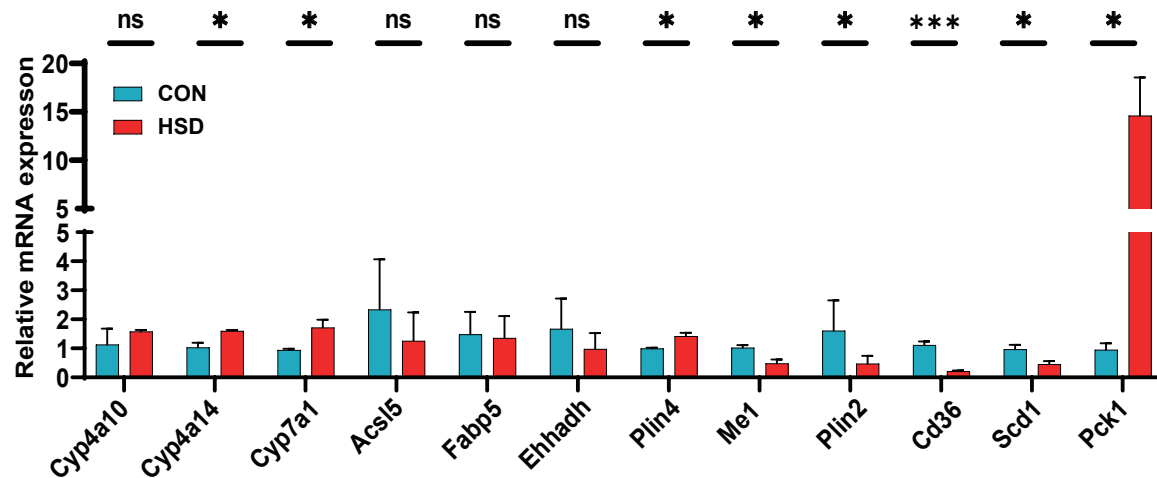

## C Bile secretion

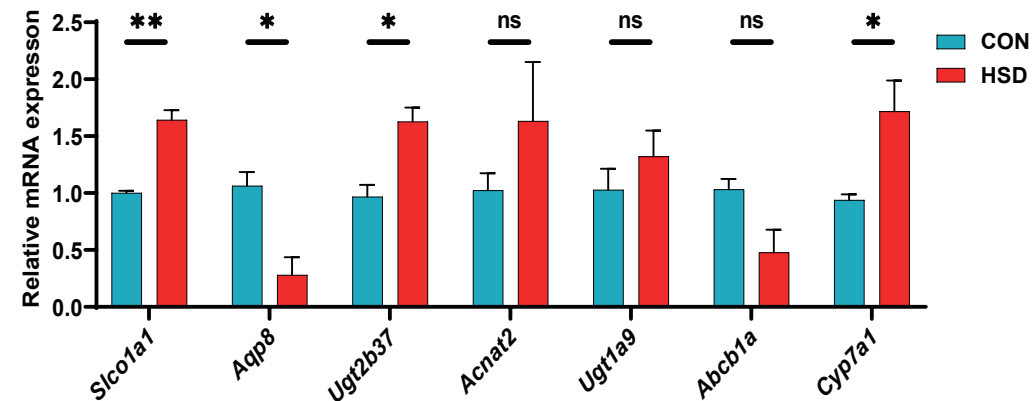

## B Retinol metabolism

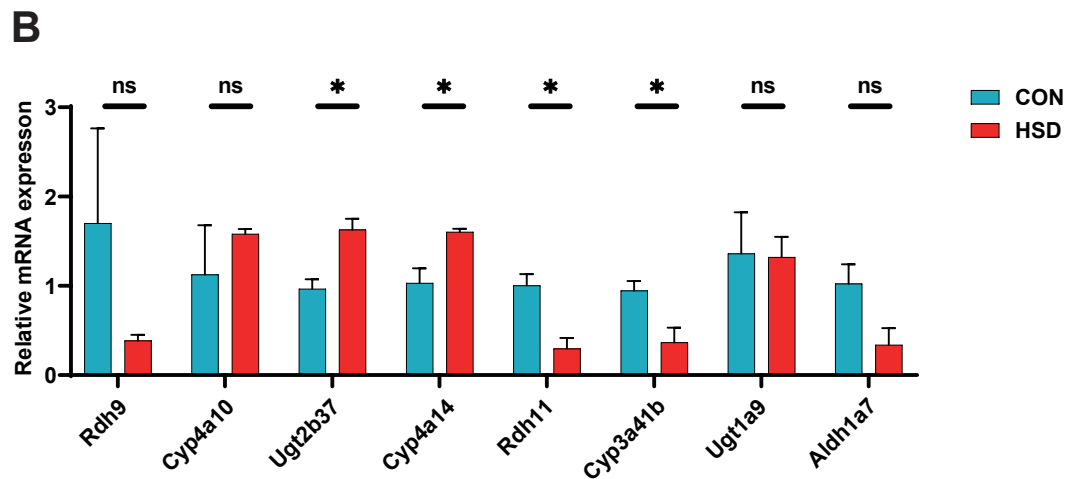

## D Cholesterol metabolism

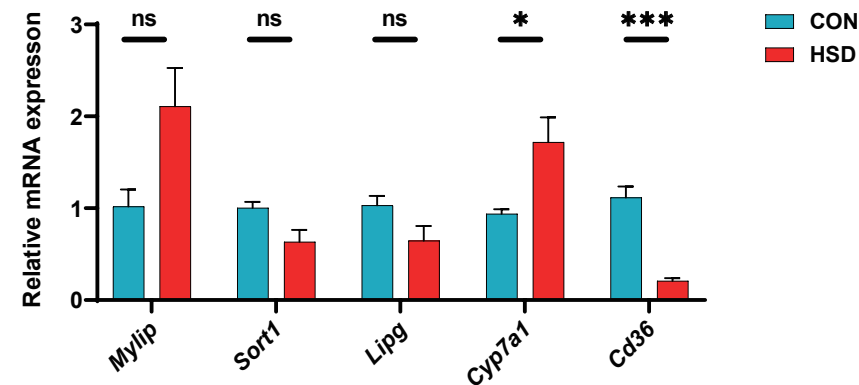

Supplement: Supplementary file 1 [file genes-14-00966-s001.zip › Supplementary Figure S6_Verification of canonical metabolic pathways.pdf]
